# One-Step Nucleic Acid Amplification in Breast Cancer Sentinel Lymph Node: A Single Institutional Experience and a Short Review

**DOI:** 10.3389/fmed.2015.00037

**Published:** 2015-06-04

**Authors:** Tatiana Brambilla, Barbara Fiamengo, Corrado Tinterri, Alberto Testori, Massimo Maria Grassi, Amedeo Sciarra, Tommaso Abbate, Wolfgang Gatzemeier, Massimo Roncalli, Luca Di Tommaso

**Affiliations:** ^1^Pathology Unit, Humanitas Clinical and Research Hospital, Milan, Italy; ^2^Senology Unit, Humanitas Clinical and Research Hospital, Milan, Italy; ^3^Senology Unit, Humanitas Gavazzeni, Bergamo, Italy

**Keywords:** breast cancer, sentinel node examination, molecular analysis, morphological analysis, permanent sections, frozen section, accuracy

## Abstract

Sentinel lymph node (SLN) examination is a standard in breast cancer patients, with several methods employed along its 20 years history, the last one represented by one-step nucleic acid amplification (OSNA). The latter is a intra-operative molecular assay searching for CK19 mRNA as a surrogate of metastatic cells. Our 3 years experience with OSNA (1122 patients) showed results overlapping those recorded in the same institution with a morphological evaluation (930 patients) of SLN. In detail, the data of OSNA were almost identical to those observed with standard post-operative procedure in terms of patients with positive SLN (30%) and micrometastatic/macrometastatic involvement of SLN (respectively, 38–45 and 62–55%). By contrast, when OSNA was compared to the standard intraoperatory procedure, it was superior in terms of accuracy, prompting the use of this molecular assay as a very valid, and reproducible for intra-operative evaluation of SLN. Further possibilities prompting the use of OSNA range from adhesion to quality control programs, saving of medical time, ability to predict, during surgery, additional nodal metastasis, and molecular bio-banking.

## Background

Sentinel lymph node (SLN) evaluation is a standard staging procedure in breast cancer patients with clinically negative axilla (1–4). Recent studies have suggested that patients with micro-metastatic SLN can avoid complete nodal dissection (5–7). By contrast, a conservative surgical approach of patients with macrometastatic SLN is highly debated and still unaddressed (8–10). Great effort is thus requested to pathologists in the evaluation and quantification of the tumor burden in SLN.

Several methods have been employed to examine SLN in a 20-year long history, all based on two different approaches. The first one is an improvement of the standard histopathological examination: SLN is evaluated using consecutive post-operatory permanent sections (PO-PS) taken from formalin-fixed paraffin-embedded tissue (1). However, the number of sections analyzed is very variable, up to specimen exhaustion (11). This approach guarantees an optimal morphological detail and it can be further supplemented by additional studies (immunohistochemical analysis, case sharing, etc.). An obvious limitation of this method is that sentinel-positive patients are subjected to a second surgical intervention. To overcome this drawback, an intraoperatory examination using frozen sections (IO-FS) of SLN (12) was introduced. However, the latter method may be limited by a lower accuracy (13), by the intraoperatory time dedicated to the procedure, and by the increased need of technical and medical resources. Also, the use of additional tools, such as imprint cytology or rapid immunohistochemistry, is not standardized and reliable enough to fill the accuracy gap between IO-FS and PO-PS (14, 15). Finally, both IO-FS and PO-PS SNL examination are characterized by a partial examination of the whole node. Indeed, the great majority of the sections are eliminated through the cutting procedure.

A completely different methodological approach is the search of a molecular fingerprint as surrogate marker of metastatic cells. Earlier studies suggested the use of cytokeratin 19 (CK19), mammaglobin, and maspin (16, 17). Indeed, when the above molecular analyses were compared to extensive morphologic examination of SLN, their sensitivity was >90% with almost absolute specificity. Accordingly, a molecular assay named one-step nucleic acid amplification (OSNA, Sysmex Corporation, Kobe, Japan) was proposed, validated, and standardized in the examination of SLN in patients affected by breast cancer (18–20). OSNA quantifies the number of CK19 mRNA copies in the whole nodal homogenate. Table S1 in Supplementary Material illustrates the comparison of criteria used to evaluate SLN status by this method and by that based on the tumor size resulting from the TNM staging system.

In this paper, we report our 3 years experience with OSNA in patients affected by breast cancer and we discuss the diagnostic performance with costs analysis, turnaround times (TATs), advantages, and limitations of this molecular assay.

## OSNA Molecular Assay at Humanitas Cancer Center

Since the introduction of OSNA, 1122 patients underwent SLN procedure at our Institution, with the number of examined nodes ranging between 1 and 4 per individual patient (mean: 1.2). A positive (metastatic) SLN was seen in 322 (29%) patients. The nodal disease was classified as 2+, macrometastasis, in 177 cases (55%) and 1+, micrometastsis, in 145 cases (45%). Results are reported in Table 1.

**Table 1:** **SLN status as evaluated by OSNA in patients affected by breast cancer**.

	OSNA first year (no. 437 pts)	OSNA second year (no. 366 pts)	OSNA third year (no. 319 pts)	OSNA (total) (no. 1122 pts)
**SLN status**
Negative	318 (73%)	263 (72%)	219 (69%)	800 (71%)
Positive	**119 (27%)**	**103 (28%)**	**100 (31%)**	**322 (29%)**
2+	81	44	52	177
1+	38	59	48	145

*Bold font highlights positive cases*.

As shown in Table 2, the vast majority of SLN 2+ patients underwent a subsequent complete axillary lymph-node dissection (ALND) where 76 (43%) patients were found to have one or more additional metastatic nodes. Among the group of SLN 1+ 97 patients underwent ALND with a significantly lower proportion of cases (15, 15%) showing additional metastasis.

**Table 2:** **Patients with additional nodal metastasis in the axillary dissection after a positive OSNA report**.

	First year	Second year	Third year	Total
Patients with additional nodal mets (after SLN 2+)	35/81 (43%)	21/42 (50%)	20/52 (38%)	76/175^a^ (43%)
Patients with additional nodal mets (after SLN 1+)	6/36 (17%)	6/41 (15%)	3/20 (15%)	15/97^a^ (15%)

*^a^2 patients after 2+ and 48 after 1+ refused complete nodal dissection following data from Galimberti et al. (5)*.

Of 91 patients with additional metastasis after complete axillary dissection, 23 (25%) showed >3 metastatic nodes; more specifically 22/23 cases had a previous OSNA report of SLN 2+ with a single case scoring 1+.

Overall, data generated by OSNA were then compared to our previous experience with SLN examination using either PO-PS or IO-FS (21). No other variables except the change in the technique affected this study because the teams involved (Pathology, Surgery, and Nuclear Medicine) were the same. Results are illustrated in Table S2 in Supplementary Material. Among the three series, the accuracy in the detection of metastatic nodes was significantly better when using PO-PS (30%) and OSNA (29%) as compared to IO-FS (22%) (*p* = 0.01). Similarly, the accuracy rate in the detection of macro- and micro-metastasis using IO-FS was significantly different as compared to other techniques (*p* < 0.01). These results may be explained by the more standardizable and reproducible protocols of OSNA and PO-PS techniques as compared to IO-FS. Indeed, the latter method is intrinsically more dependent on the experience of the operator (thickness and number of sections, integrity of sections, manual ability of the technician, etc.).

The predictive value of additional nodal mets of a positive sentinel node in patients with axillary dissection showed more homogeneous data among the three techniques. The values ranged between 31 and 33%, with OSNA and PO-PS series showing again the most overlapping results. OSNA 2+ and macromets yielded, as expected, a higher number of additional mets in the axillary dissection, as compared to OSNA 1+ and micromets.

## OSNA Molecular Assay: Literature Overview

The reliability of the OSNA assay has been repeatedly demonstrated in the literature. High specificity, sensitivity, accuracy, positive, and negative predictive values have validated OSNA as a very good and objective method to investigate the SLN status in breast cancer patients (22). As shown in Table S3 in Supplementary Material, some differences have been reported as to the rate of positive nodes and the distribution of OSNA scores (–, 1+, 2+), likely related to differences in the series. For example, an accurate clinical, radiological, and cytological pre-operative evaluation of the axilla may permit a diagnosis of metastatic node (macrometastasis), which is not further subjected to the molecular assay, thus increasing the relative number of OSNA cases scoring 1+. However, all the studies report similar results in the percentage of additional nodal mets, with values close to 40% for macromets and to 16% for micromets.

## Turnaround Time and Cost Analysis

The average TAT per single procedure was 48 min. for OSNA and 50 min for IO-FS. More specifically, the allocated time for the pathologist was 5 min for OSNA and 45 min with IO-FS. The allocated time for the technician was similar in both the procedures (50 min). In terms of consumables and equipment OSNA were more expensive than IO-FS while in terms of human resources IO-FS were more expensive than OSNA. In keeping with other reports based on the molecular evaluation of SLN (23, 24), during the first year, we annotated a cost of 270€ for a single procedure.

During the first year of OSNA experience, all SLNs were evaluated regardless the number of scheduled procedures or whether the node was macroscopically metastatic. During the second and third year, we decided to perform the molecular assay if at least four patients were scheduled for SLN procedure and never in cases of grossly metastatic nodes. This means that we performed IO-FS whenever up to three SNL procedures were scheduled (mixed regimen). Indeed, a retrospective cost analysis showed us that the OSNA procedure for a single SLN/day was much more expensive than the same single procedure conducted in a surgical session with four or more scheduled SLNs. This is because a fixed charge is due for the activation of the OSNA equipment. As a consequence of this selection policy in the use of the molecular assay, our costs were significantly reduced. However, cases with up to three procedures/day were conducted using IO-FS with a negative impact on the medical time allocated to the procedure (up to 3 h/day).

Also, interesting is the cost comparison between OSNA and SNL examination conducted on permanent sections. Two studies coming from the UK, one conducted in a large district general hospital (23) and the other by the University and the Health Economic Consortium of York (25) showed that the savings in terms of reduced secondary surgeries and bed occupancy, could potentially be 150€ per patients.

## Advantages and Disadvantages

Provided that OSNA is more standardized than IO-FS and at least equally accurate, the most significant advantage is the medical time saved during the procedure. From a practical point of view, having adopted OSNA as the method to examine SLN when at least four procedures are scheduled, pathologists in charge will save at least 2.5 h during each surgical session. Having had sessions up to nine procedures, this meant a full day of a working pathologist. A further advantage is the possibility to perform in the same introperatory time a IO-FS and a OSNA procedure, provided to have two technicians, a single pathologist, a cryostat and a OSNA equipment. A third aspect is the possibility to evaluate more than one SLN (up to four) in the same run. This might even contemplate the possibility to cumulate nodes from different patients; this is quite unusual at least in our experience but it may occur in centers with multiple synchronous surgical breast procedures. A more common occurrence (about 10% of the cases in our series) is the synchronous examination of more than one node for a single patient. Needless to say, such a condition is not feasible with a IO-FS approach. Finally, the post-OSNA residual node homogenate is a bank of biological molecules and a potential source of material for future investigations. Last, but not least, a quality control program can be easily undertaken among labs using this molecular technique.

Among disadvantages, cost and TAT have been already discussed. The existence of CK19 negative breast tumor may have a negative impact on the specificity of the OSNA procedure. This possibility was estimated to occur in 2–3% of breast cancer evaluated using immunohistochemistry. However, Pegolo et al. (26) showed that cases with negative CK19 immunostaining had a significant CK19 expression at mRNA level. We have also adopted and recommend the use of intra-operative cytological smear before starting an OSNA procedure to avoid false-negative cases. Benign epithelial inclusions and epithelial cells displacements might also be the cause of false-positive results (27, 28); however, the low levels of CK19 mRNA copies in these particular cases are reported to be below the cutoff of an OSNA positive test. However, in IO-FS, these inclusions/cell displacements might be very difficult to be differentiated from micro-metastasis or isolated tumor cells, as well as from intra-capsular nevi.

## Tumor Burden and Prediction of Axillary Status

One of the most exciting advantage of OSNA over conventional IO-FS examination of SLN is that the former provides a value in terms of mRNA copy number (representative of the tumoral burden), which can been used to predict additional mets in ALND. Importantly, several investigators have already developed nomograms associated with ALND mets. However, these nomograms can be applied after the histological examination of breast cancer (29) and not during the intraoperatory time. By contrast, OSNA-based nomograms (30, 31 and Di Filippo, personal communication) may be used during the intraoperatory time and as such data they can be immediately used to re-orient the surgical management of the case (to perform or not the axillary dissection).

## Conclusion

The molecular evaluation of SLN in breast cancer patients appears as a frontier currently explored by pathologists. When we were firstly exposed to this technique our major concern was to perform a diagnosis without the fundamental aid of the microscope nor of the human eye. However, after 3 years of experience, we are much more confident in the technique which showed reproducible results over 3 years in more than 1000 SLN analyzed. Importantly, when OSNA was compared to the other standard intraoperatory procedure (IO-FS), it was superior in terms of accuracy. We therefore endorse OSNA as a very valid and reproducible assay for SLN.

The molecular evaluation of SLN can be very fruitful. Standardization and reproducibility are very important issues in a pathology lab and they can be easily and quickly obtained. The possibility to adhere to quality control programs is another not negligible opportunity. In addition, the saving of medical time is also and obviously especially important. Further, golden opportunities of this frontier are to be expected. The possibility to predict, during surgery (32–36), additional nodal mets would address the critical issue raised by the ACOSG Z0011 study (8); while mRNA analysis using gene expression from metastatic foci would hopefully provide predictive information on the tumor behavior.

## Conflict of Interest Statement

The authors declare that the research was conducted in the absence of any commercial or financial relationships that could be construed as a potential conflict of interest.

## Supplementary Material

The Supplementary Material for this article can be found online at http://journal.frontiersin.org/article/10.3389/fmed.2015.00037/abstract
