# Supplementary material for: One-Step Nucleic Acid Amplification in Breast Cancer Sentinel Lymph Node: A Single Institutional Experience and a Short Review
Source: Front Med (Lausanne). 2015 Jun 4;2:37. doi: 10.3389/fmed.2015.00037 (PMC4469115; doi:10.3389/fmed.2015.00037)
Supplement: Supplementary file 1 [file table_1.pdf]

Supplementary Table 1. A comparison between OSNA- and TNM-based criteria with corresponding diagnostic categories.

**OSNA-based criteria (CK19 mRNA copies/µL)**	**TNM-based criteria (size of metastatic focus)**	**Daignostic categories of SLN status OSNA / TNM**
≥5000	>2 mm	2+ / macrometastasis
250-4999	0,2-2 mm	1+ / micrometastasis
<250	<0,2 mm	- / negative
