# Supplementary material for: One-Step Nucleic Acid Amplification in Breast Cancer Sentinel Lymph Node: A Single Institutional Experience and a Short Review
Source: Front Med (Lausanne). 2015 Jun 4;2:37. doi: 10.3389/fmed.2015.00037 (PMC4469115; doi:10.3389/fmed.2015.00037)
Supplement: Supplementary file 2 [file table_2.pdf]

Supplementary Table 2. Performance of morphological (PO-PS and IO-FS) and molecular (OSNA) techniques in SLN evaluation. Experience at Humanitas Cancer Center.

	**PO-PS**	**IO-FS**	**OSNA**
**Number of patients**	**540**	**390**	**1122**
**Patients with positive SLN**	**162 (30%)**	**87 (22%)**	**322 (29%)**
- macromets<br>- OSNA 2+	100 (62%)	64 (74%)	177 (55%)
- micromets<br>-OSNA 1+	62 (38%)	23 (26%)	145 (45%)
**Patients with additional nodal mets after ALND**	**53/162 (33%)**	**27/87 (31%)**	**91/272* (33%)**
- macromets<br>- OSNA 2+	43/100 (43%)	23/64 (36%)	76/175 (43%)
- micromets<br>- OSNA 1+	10/62 (16%)	4/23 (17%)	15/97 (15%)

*2 patients after 2+ and 48 after 1+ refused complete nodal dissection following data from Galimberti et al (5).
