# Supplementary material for: One-Step Nucleic Acid Amplification in Breast Cancer Sentinel Lymph Node: A Single Institutional Experience and a Short Review
Source: Front Med (Lausanne). 2015 Jun 4;2:37. doi: 10.3389/fmed.2015.00037 (PMC4469115; doi:10.3389/fmed.2015.00037)
Supplement: Supplementary file 3 [file table_3.pdf]

Supplementary Table 3. Rate of positive SLN, distribution of 2+ and 1+ cases, and their impact on nodal mets after complete axillary dissection: data from largest OSNA studies.

**Reference**	**Cases**	**Positive SLN**	**++**	**+**	**ALND involvement in pts with SLN 2+**	**ALND involvement in pts with SLN 1+**
Deambrogio et al (22)	1080	22%	49%	51%	47%	18%
Teramoto et al (23)	833	21%	60%	40%	43%	11%
Babar et al (24)	859	31%	47%	38%*	35%	16%
Espinosa et al (25)	306	35%	56%	41%*	37%	18%
Present series	1122	29%	55%	45%	43%	15%

* these data did not include diluted samples, separately reported in these papers.
